# Inhibition of Dengue Virus Entry and Multiplication into Monocytes Using RNA Interference

**DOI:** 10.1371/journal.pntd.0001410

**Published:** 2011-11-29

**Authors:** Mohammed Abdelfatah Alhoot, Seok Mui Wang, Shamala Devi Sekaran

**Affiliations:** Department of Medical Microbiology, Faculty of Medicine, University of Malaya, Kuala Lumpur, Malaysia; Texas Biomedical Research Institute, United States of America

## Abstract

**Background:**

Dengue infection ranks as one of the most significant viral diseases of the globe. Currently, there is no specific vaccine or antiviral therapy for prevention or treatment. Monocytes/macrophages are the principal target cells for dengue virus and are responsible for disseminating the virus after its transmission. Dengue virus enters target cells via receptor-mediated endocytosis after the viral envelope protein E attaches to the cell surface receptor. This study aimed to investigate the effect of silencing the CD-14 associated molecule and clathrin-mediated endocytosis using siRNA on dengue virus entry into monocytes.

**Methodology/Principal Findings:**

Gene expression analysis showed a significant down-regulation of the target genes (82.7%, 84.9 and 76.3% for CD-14 associated molecule, CLTC and DNM2 respectively) in transfected monocytes. The effect of silencing of target genes on dengue virus entry into monocytes was investigated by infecting silenced and non-silenced monocytes with DENV-2. Results showed a significant reduction of infected cells (85.2%), intracellular viral RNA load (73.0%), and extracellular viral RNA load (63.0%) in silenced monocytes as compared to non-silenced monocytes.

**Conclusions/Significance:**

Silencing the cell surface receptor and clathrin mediated endocytosis using RNA interference resulted in inhibition of the dengue virus entry and subsequently multiplication of the virus in the monocytes. This might serve as a novel promising therapeutic target to attenuate dengue infection and thus reduce transmission as well as progression to severe dengue hemorrhagic fever.

## Author Summary

Prevention and treatment of dengue infection remain a serious global public health priority. Extensive efforts are required toward the development of vaccines and discovery of potential therapeutic compounds against the dengue viruses. Dengue virus entry is a critical step for virus reproduction and establishes the infection. Hence, the blockade of dengue virus entry into the host cell is an interesting antiviral strategy as it represents a barrier to suppress the onset of infection. This study was achieved by using RNA interference to silence the cellular receptor, and the clathrin mediated endocytosis that enhances the entry of dengue virus in monocytes. Results showed a marked reduction of infected monocytes by flow cytometry. In addition, both intracellular and extracellular viral RNA load was shown to be reduced in treated monocytes when compared to untreated monocytes. Based on these findings, this study concludes that this therapeutic strategy of blocking the virus replication at the first stage of multiplication might serve as a hopeful drug to mitigate the dengue symptoms, and reduction the disease severity.

## Introduction

Dengue infection ranks as one of the most clinically significant and prevalent mosquito-borne viral diseases of the globe. It is an expanding public health problem particularly in the tropical and subtropical areas [1]. Following an incubation period of 3 to 14 days, fever and a variety of symptoms occur, coinciding with the appearance of dengue virus (DENV) in blood [2]. Immunopathological studies suggest that many tissues may be involved during dengue infection, as viral antigens are expressed in liver, lymph node, spleen and bone marrow [3], [4], [5]. DENV can infect and replicate in different mammalian cells, including monocytes, macrophages, dendritic cells, B and T leukocytes, endothelial cells, and bone marrow-, hepatoma-, neuroblastoma- and kidney-derived cells. Based on several observations and antibody dependent enhancement hypothesis, monocyte lineage cells are the major target for DENV [6], [7], [8], [9]. These cells are responsible for replication and dissemination of the virus after the infection from mosquito bites. Since monocytes/macrophages are active phagocytic cells with cytoplasmic lysosomal components that can eliminate microorganisms [10], the interaction of DENV with monocytes/macrophages may have detrimental effects on both virus and cells. DENV infected monocytes/macrophages release soluble mediators that strongly influence the biological characteristics of endothelial cells and the hematopoietic cell population. This indicates that the interactions between DENV and monocytes/macrophages are important in the pathogenesis of dengue hemorrhagic fever (DHF) and dengue shock syndrome (DSS).

Previous studies suggest that DENV enters target cells after the viral envelope protein E attaches to an uncharacterized cell receptor [11]. Current studies indicate that multiple cell surface molecules, including GRP78 [12], heat shock proteins (Hsp) 70 and 90 [13], [14], lipopolysaccharide-binding CD 14-associated molecule [8], [15], [16], laminin receptor [17], mannose receptor [18], and DC-SIGN [19], were involved in DENV binding and subsequent virus infection in different target cells. CD-14 associated molecule has implicated as a surface receptors on monocytes for DENV-2 entry [8], [15], [16]. CD-14 associated molecule is a membrane protein expressed by monocytes, antigen presenting cells and neutrophils and plays a role in the innate immune system. CD-14 associated molecule is necessary for the cellular response in infections mediated by bacterial lipopolysaccharide, which activates monocytes for the expression of cytokines, growth factors, and procoagulatory factors [20]. Failure of interaction of lipopolysaccharide with CD-14 has been implicated in susceptibility to infectious diseases [21].

After binding to the surface receptor, DENV internalizes into the cell cytoplasm by membrane fusion and this process may take place within intracellular vesicles (pH-dependent). This endocytic internalization offers the advantage of guiding the virion to an adequate site for replication and bypassing many cytoplasmatic barriers. Several endocytic pathways have recently been identified [22], but the clathrin-mediated endocytosis has been demonstrated as the main entry pathway for DENV-2 [23], [24], [25]. Clathrin-mediated endocytosis plays a crucial role in the formation of coated vesicles, antigen presentation, nutrient acquisition, clearance of apoptotic cells, pathogen entry, receptor regulation, hypertension, and synaptic transmission.

Despite the importance and increasing incidence of DENV as a human pathogen, there are no antiviral agents or vaccines available for treatment or prevention. It is necessary to develop new therapeutic strategies by targeting the DENV binding molecules on susceptible host cells. The blockade of DENV entry into the host cell is an interesting antiviral strategy because it represents a barrier to suppress the onset of infection [26].

RNA interference (RNAi) is a potent and specific post-transcriptional gene silencing event induced by double stranded RNAs, which degrade the target mRNA that has the homologous sequence [27], [28]. RNAi has become the most useful and powerful research tool for studies of gene functions, regulation of gene transcriptions and gene therapies over the last few years [29]. Compared to other traditional gene silencing methods, it has the advantage of significantly enhanced potency, specificity and versatility [30], [31]. The mediators of sequence specific mRNA degradation are small interfering RNAs (siRNA). siRNAs are 21 to 29 nucleotide in length and carry 5′ phosphate and 3′ hydroxyl termini and 2-nt 3′ overhangs [32], [33]. siRNAs can effectively suppress target genes in mammalian cells without triggering interferon production [30], [34]. The transfection of siRNAs into mammalian cells resulted in a potent, long-lasting reaction typically several days and extraordinarily specific post-transcriptional silencing of target genes [33], [35]. The use of siRNA is suitable for the design of novel gene-specific therapeutics by directly targeting the mRNAs of the related genes and holds great promise for the application of gene-specific therapies in treating acute diseases such as viral infection, cancer, and, perhaps, acute inflammation. Several preliminary studies show that, RNAi has been used effectively to inhibit the replication of different pathogenic viruses in culture, including respiratory syncytial virus, hepatitis viruses, influenza virus, poliovirus, rhinovirus and human immunodeficiency virus [30], [35].

This study aimed to investigate the possible usage of siRNA in the reduction of DENV entry and replication in human monocytes by targeting the monocytes attachment receptor and clathrin-mediated endocytosis. CD-14 associated molecule has been targeted as an attachment receptor on the surface of monocytes. Besides that, two main components of the clathrin-mediated endocytosis pathway has also been selected; Clathrin heavy polypeptide (CLTC) which is required for clathrin-coated pits formation, and Dynamin 2 (Human-DNM2) which is important for pinching off of endocytic vesicles from the plasma membrane. Three siRNAs were designed for each target gene (CD-14 associated molecule, CLTC, and DNM2) to guarantee a greater potency than a single siRNA and eliminate off-targets effect.

## Methods

### Isolation and purification of monocytes

Peripheral blood samples from different healthy donors who were not associated with prior dengue infection were collected in EDTA tubes. Peripheral blood mononuclear cells (PBMC) were isolated by density centrifugation with Ficoll-paque as described previously [36]. Human monocytes were purified by depletion of non-monocytes followed by negative selection using a MACS human monocytes isolation kit II (cat #: 130-091-153, Miltenyi Biotec GmbH, Gladbach, Germany) in accordance to manufacturer's protocol. Briefly, non-monocytes were indirectly magnetically labeled with a cocktail of biotin-conjugated mouse monoclonal antibodies against CD3, CD7, CD16, CD19, CD56, CD123 and Glycohorin A, as primary labeling reagents, and anti-biotin monoclonal antibodies that were conjugated to MicroBeads, as secondary labeling reagents. The magnetically labeled non-monocytes were depleted by retaining them on a MACS® column in the magnetic field of a MACS separator, while the unlabeled monocytes passed through the column. Written informed consent was obtained from blood donors. Ethical clearance (REF No.: 794.5) was obtained for the main project from the Scientific and Ethical Committee at the University Malaya Medical Center prior to commencement of study.

### siRNA sequences

The nucleotide sequences for the CD-14 associated molecule (NM_000591), CLTC (NM_004859), and DNM2 (NM_001005360) transcripts were obtained from GenBank. siRNA sequences were designed by using the web-based tool IDT SciTools RNAi Design available from Integrated DNA Technologies (IDT), Inc. at www.idtdna.com. Three siRNAs were designed for each gene (Table 1). A pool consists of the three siRNAs for each gene was custom chemically synthesized by 1^st^ BASE Pte Ltd, Singapore. The synthesized siRNAs were purified by HPLC, and a 2′-O-methyl modification at position 2 was introduced to deactivate the off-target activity of the siRNA without compromising the silencing effectiveness [37].

**Table 1:** Sequences of siRNA oligonucleotide template.

siRNA	Nucleotide sequence (sense strand)	Position^a^
**CD-14 (1)**	5′r(CUGCAGAAUCCUUCCUGUUACGGTC)3′	72–96
**CD-14 (2)**	5′r(CCUCUGGAAGCCACAGGACUUGCAC)3′	760–784
**CD-14 (3)**	5′r(ACAGAAUAAUGAAUGGACUCAAACT)3′	1491–1515
**CLTC (1)**	5′r(AGCCAGGACCCAGAUGUFC)3′	2607–2625
**CLTC (2)**	5′r(AUGUAUGAUGCUGCUAAGU)3′	4071–4089
**CLTC (3)**	5′r(CUCCACCAAUGACCUUAGA)3′	7964–7982
**DNM2 (1)**	5′r(GAAGGACAUCCGUGCAGCACUGGCA)3′	925–949
**DNM2 (2)**	5′r(GUACCAGUAAGCUCAGUUCCUACCC)3′	1515–1539
**DNM2 (3)**	5′r(CCCUUGACACCAUCCUGAAUGAGGG)3′	3075–3099
**β- actin**	5′r(CCAGCACAAUGAAGAUCAAGAUCAT)3′	1049–1073
**Scramble** b	5′r(ATGGACAGAATAAATGGACTT)3′	

a The position refers to the siRNA oligonucleotide position on the target gene.

b Scrambled siRNA is a control used to discount any changes to the gene expression profile that may result from the siRNA delivery method.

Three siRNAs for each target gene were designed. The experiment includes also one Positive control siRNA for human β-actin gene and one scramble siRNA oligonucleotide.

### Transfection of monocytes with siRNA

The siRNAs were transfected using siLentFect™ Lipid Reagent (cat #: 170-3361, Bio-Rad Laboratories, Hercules, CA, USA). Transfection was achieved at a cell density of 1.5×10^5^ cells/well in a 24 wells plate by using 1.0 µl of lipid transfection reagent and optimized siRNA concentration (25, 50, and 50 nM for CD14 associate molecule, CLTC, and DNM2), respectively. The positive control was transfected with pre-validated siRNA oligonucleotide targeting β-actin gene. The negative control received the siLentFect™ Lipid Reagent plus the scramble siRNA oligonucleotides. After 24 h, the cells were harvested for monitoring the gene silencing effect at the mRNA expression level while the infectious entry was evaluated after infecting the transfected monocytes with DENV-2 after 72 h.

### Cell viability and Cytotoxicity

Cytotoxicity was tested by a fluorescent assay that measure LDH released from cells with compromised membrane using CytoTox-ONE™ Homogeneous Membrane Integrity Assay (Promega, Madison, WI) in accordance to manufacturer's protocol. Viable monocytes also were counted by staining with 0.4% trypan blue (Invitrogen). The number of trypan blue positive and negative cells were counted on a hemocytometer under light microscope. Cell counts were used to verify the LDH results.

### RNA extraction

Total RNA was extracted using RNeasy® Plus Micro Kit (cat #: 74034, QIAGEN, Hilden, Germany) for gene expression analysis and intracellular DENV quantification by reverse transcription quantitative real-time PCR (RT-qPCR). RNA extraction was done according to the manufacturer's instructions. Total RNA was purified using gDNA eliminator columns and was checked by including no-reverse transcription control in the RT-qPCR amplification procedure. For quantification of DENV in the cellular supernatant, viral RNA was extracted separately from 200 µl of tissue culture supernatant using the QIAmp viral RNA mini kit (cat #: 52904, QIAGEN, Hilden, Germany) according to the manufacturer's specifications.

All RNA samples were examined for their purity and concentration using Implen NanoPhotometer™ (Implen GmbH, Munich, Germany). All extracted samples have absorbance ratios of 1.8–2.0 at 260/280 nm, indicating all the samples were free from potentially accumulating proteins during the RNA extraction procedure. In addition, the absence of RNA degradation was assessed by agarose gel electrophoresis. Isolated RNA was stored at −80°C until used.

### cDNA synthesis

For gene expression analysis, 500 ng of total RNA were reverse-transcribed into cDNA using the iScript™ cDNA synthesis kit (cat #: 170-8891, Bio-Rad Laboratories, Hercules, CA, USA).

### Optimization of qPCR

All sequences of target gene [CD-14 (CD-14 associated molecule); CLTC (Clathrin heavy polypeptide); and DNM2 (Dynamin 2 )], experimental control gene [ACTB (β-actin)] and reference genes [RPL22 (ribosomal protein L22); and RPS29 (ribosomal protein S29)] were retrieved from GenBank. qPCR primer pairs for CD-14, CLTC, DNM2, and ACTB were designed by using Primer Express software V3.0. Primers for RPL22 and RPS29 were obtained from published sequences [38]. Various concentrations of primer sets and a range of annealing temperatures were analyzed to achieve optimal qPCR specificity and efficiency (data not shown). A dissociation analysis was performed after each qPCR runs with cDNA as a template, in order to show that each primer set amplified the expected single product. A pair of primer was considered valid when the efficacy of amplification is between 90–110% with a minimum r^2^ of 0.980. Table 2 shows the primers used in this study. We used the geNorm algorithm v3.5 [39] to determine the number and the most stable reference genes for this study (data not shown).

**Table 2:** Primer sequences.

Accession Number	Gene symbol	Sequence (5′→3′)	(bp)
**( NM_000591)**	CD-14	F: GACAGGGCGTTCTTGGCTCGR: ACAGAGAGCCGCCATCAGTCC	187
**(NM_004859)**	CLTC	F: CCACAATACTCCACCAATGACCTTAR: CCCATTTTAAGTGGCTCCACCTCTC	97
**(NM_001005360)**	DNM2	F: GGCATTCGAGGCCATTGTR: CATTTCAGACAGGGCTCTTTCA	60
**(NM_001101)**	ACTB	F: CCTTCTACAATGAGCTGCGTGTGR: AGGTAGTCAGTCAGGTCCCG	301
**(NM_000983)**	RPL22	F: TCGCTCACCTCCCTTTCTAAR: TCACGGTGATCTTGCTCTTG	250
**(NM_001030001)**	RPS29	F: GCACTGCTGAGAGCAAGATGR: ATAGGCAGTGCCAAGGAAGA	213
**(NC001477)**	DENV_NS5	F: GGAAGGAGAAGGACTGCACAR: ATTCTTGTGTCCCATCCTGCT	104

Primer sequences of the target genes, positive control gene, optimal reference genes and DENV NS5.

### qPCR and gene expression

qPCR was performed on CFX96™ Real-Time PCR Detection System (Bio-Rad, Hercules, CA, USA). The reaction was carried out in a final volume of 20 µl, including 10 µl 2× iQ™ SYBR® Green Supermix (cat #: 170-8882, Bio-Rad Laboratories, Hercules, CA, USA), 2 µl cDNA template, 6 µl RNase/DNase-free sterile water and 1.0 µl (250 nM final concentration) of each primer. qPCR was carried out with an initial 5 min hot start activation of the polymerase at 95°C then 40 cycles of 10 sec denaturation at 95°C, 20 sec annealing at 57.7°C with a single fluorescence emission measurement and 30 sec extension at 72°C, followed by 5 min at 72°C for final extension. The specificity of amplicons was verified by melting curve analysis (74 to 95°C) with a heating rate 0.5°C per 5 sec to verify the identity and purity of the amplified products. qPCR experiments were performed in triplicate. Also, no template and no-reverse transcription controls were included. Baseline and quantification cycle (Cq) values and gene expression analysis were done using the Bio-Rad CFX Manager Software 1.6.

### Virus propagation and titration

DENV-2 New Guinea Clone (NGC) stock was propagated in C6/36 cells and stored at −80°C until used as described previously [40]. Virus was titrated using the plaque formation assay on PscloneD cells as described previously [41].

### Ex-vivo monocytes infection

Infection of silenced and non-silenced monocytes was performed in a 24-wells tissue culture plate. 1.5×10^5^ cells per well were seeded in each well. At the time of infection, medium was removed, DENV-2 (MOI of 2) was added, and followed by incubation at 37°C for 2 h to allow viral adsorption. The culture plate was gently agitated every 15 min for optimal virus to cell contact. Thereafter, the viral supernatant was removed, and the cells were washed three times with serum free media to remove residual virus. Fresh complete growth RPMI 1640 medium was added and then incubated at 37°C and 5% CO_2_ for 72 h prior to harvest. Cellular supernatant was collected and stored in aliquot at −80°C until use for viral RNA copies quantification by RT-qPCR, while monocytes were harvested for monitoring intracellular infectious DENV by flow cytometry and quantification of viral RNA copies by RT-qPCR.

### Dengue virus quantification by RT-qPCR

Quantification of dengue viral RNA by RT-qPCR requires a reliable standard curve. 10-fold serial diluted of a known copies number of DENV RNA was diluted and used to generate the standard curve. One-step RT-qPCR was carried out in CFX96™ Real-Time PCR Detection System using the iScript™ One-Step RT-PCR Kit with SYBR® Green (cat #: 170-8893, Bio-Rad Laboratories, Hercules, CA, USA). After optimization, the reaction was performed in a final volume of 25 µl, including 12.5 µl 2× SYBR® Green RT-PCR reaction mix, 6.25 µl RNase/DNase-free sterile water and 0.5 µl (200 nM final concentration) of each primer (Table 2), 0.25 µl iScript Reverse Transcriptase for One-Step RT-PCR and 5 µl RNA template. The thermal cycling profile of this assay consisted of a 30 min reverse transcription step at 50°C, 15 min of Taq polymerase activation at 95°C, followed by 35 cycles of PCR at 95°C of denaturing for 30 sec, 40 sec annealing at 58.0°C and 50 sec extension at 72.0°C with a step of a single fluorescence emission data collection followed by 10 min at 72°C for final extension. The specificity of amplicon was verified by melting curve analysis (72 to 95°C) with a heating rate 0.5°C per 5 sec to check the identity and purity of the amplified products. Triplicate reactions were carried out for each sample, and no template control was included. Cq values and number of copies for DENV-2 RNA per sample were calculated using the Bio-Rad CFX Manager Software 1.6.

### Flow cytometry analysis

Silenced and non-silenced DENV-2 infected and non-infected monocytes (control) were harvested at 72 h after DENV-2 infection for flow cytometric determination of DENV infection. Cells were washed twice in PBS by centrifugation, fixed and permeabilized in 200 µl Cytofix/Cytoperm solution (BD Biosciences, San Diego, CA) for 15 min at room temperature followed by washing twice in Cytoperm/Cytowash solution (BD Biosciences, San Diego, CA). Cells were stained using indirect staining method. First, cells were incubated in 100 µl of MAb (anti-DENV-2 for detection of positive samples and anti-DENV-3 as a negative control) as a primary antibody for 1 h on ice. The cells were then washed twice with Cytoperm/Cytowash solution and incubated with a FITC-labeled goat anti-mouse IgG (50 µl) as a secondary antibody at a final concentration of 3.5 µg/mL for 30 min on ice in the dark. Cells were subsequently washed twice with an excess amount of Cytoperm/Cytowash solution and resuspended in 0.5 ml of stain buffer (BD Biosciences, San Diego, CA). As a final point, 50,000 events were acquired on a FACScaliber using Cell Quest software (Becton Dickinson Immunocytometry System, San Diego, CA). The percentage of dengue positive cells was determined from FITC fluorescence histogram using a region that was defined based on analysis of the infected control monocytes.

### Statistical analysis

All assays (Cytotoxicity assay, gene expression analysis, monocytes infection experiment, flow cytometry detection of infected cells, and intracellular and extracellular quantification of viral RAN by RT-qPCR) were done in triplicate. All statistical analyses were performed using GraphPad Prism version 5.01 (GraphPad Software, San Diego, CA). P values<0.05 were considered significant. Error bars are expressed as ± SD.

## Results

### siRNA delivery

Our aim was to perform an efficient siRNA transfection to achieve a maximum silencing effect of the target mRNAs. We have optimized the transfection experiment to produce a maximum silencing with minimal or no toxicity to the host cells. We observed that the highest efficiency of siRNA transfection could be achieved at a cell density of 1.5×10^5^ cells/well in a 24 wells plate by using 1.0 µl of siLentFect Lipid transfection reagent. The optimal siRNA concentration was 25, 50, and 50 nM for CD14 associate molecule, CLTC and DNM2 respectively (data not shown).

### Efficiency of silencing target gene

**Figure 1:**
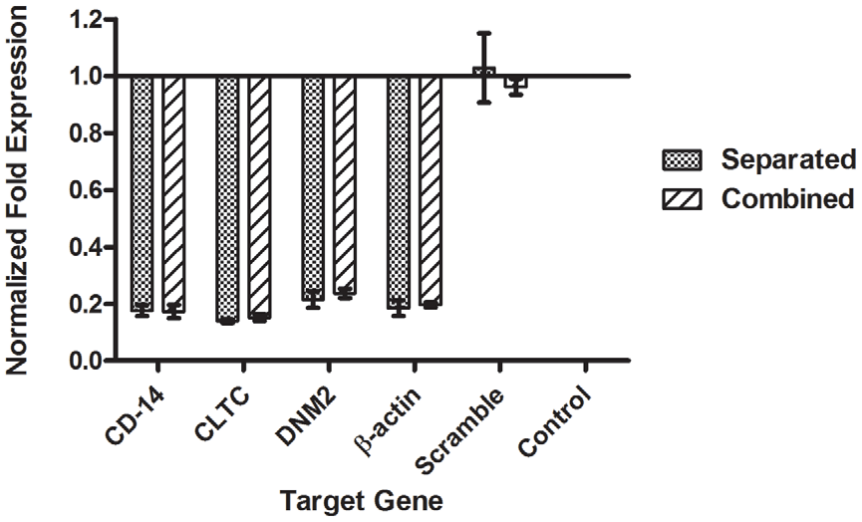
Silencing efficiency of target genes. siRNA pools were proven to be specific and potent in silencing target genes. Each gene of the target genes was targeted with a pool of siRNAs consisted of three different siRNAs. Results showed an efficient gene knockdown when compared with non-transfected control and normalized to reference genes (One-way ANOVA with Dunnett's post-test, P<0.0001, Error bars are ± SD). The effect of a combined transfection of the siRNA pools in monocytes is consistent, and there are no significant differences between separated transfection, (82.4%±1.9), (86.0%±0.6), and (78.5%±3.0), and combined transfection (82.7%±2.4), (84.9%±1.2), and (76.3%±1.7), on expression of CD-14 associated molecule, CLTC, and DNM2, respectively (Two-way ANOVA with Bonferroni post-test, P>0.05, Error bars are ± SD). Scrambled siRNA had no inhibitory effect on any gene expression and showed a similar expression to the non-transfected control.

RT-qPCR was performed with mRNA extracted from the transfected monocytes to examine the knockdown level of the target mRNAs induced by siRNA transfection. Gene expression was calculated using the algorithm provided by Bio-Rad CFX Manager Software 1.6, which is based on the algorithm outlined previously [39]. Results were expressed as normalized fold expression as compared to the non-transfected control and normalized to reference genes. First, we screened the silencing efficiency of pooled siRNAs, which comprised of three different siRNAs targeting the same gene as shown in Figure 1. The knockdown levels were 82.4%±1.9, 86.0%±0.6 and 78.5%±3.0 for pooled CD-14 associated molecule siRNA, pooled CLTC siRNA and pooled DNM2 siRNA respectively (One-way ANOVA with Dunnett's post-test, P<0.0001). The monocytes were then transfected with a combination of the three different siRNA pools to target on all the three genes simultaneously. We found that there are no significant differences in the knockdown level for all the genes (82.7%±2.4, 84.9%±1.2 and 76.3%±1.7 for CD-14 associated molecule, CLTC and DNM2 respectively) when compared to separate transfection (Two-way ANOVA with Bonferroni post-test, P>0.05). Furthermore, scramble siRNA had no inhibitory effect on any gene expression and observation was similar to the non-transfected control (Figure 1).

### Cytotoxicity testing

**Figure 2:**
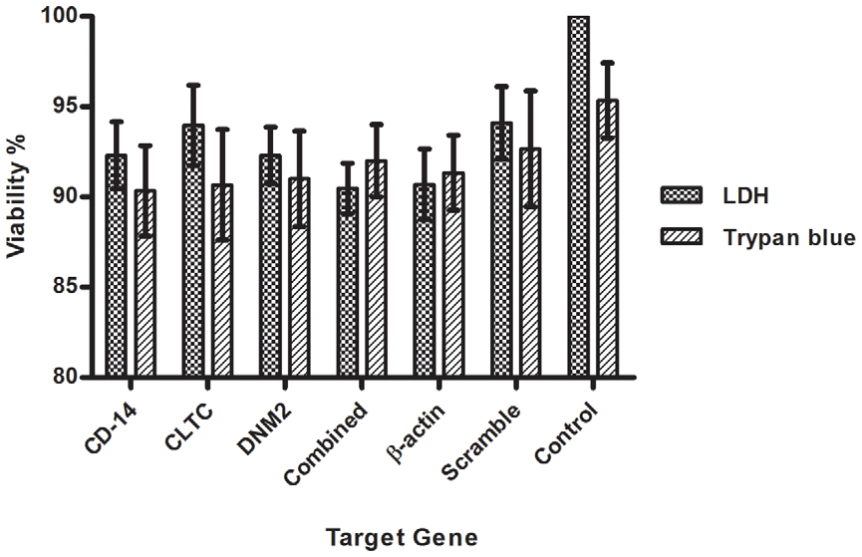
Transfection cytotoxicity. Monocytes were exposed to siRNA at a concentration of 25, 50, and 50 nM for CD-14 associate molecule, CLTC, and DNM2, respectively. Cytotoxicity was determined by measuring LDH level in the culture supernatant and by counting trypan blue stained viable cells. Results showed siRNAs are not cytotoxic (>90% live cells) for all pools of siRNA. Combined transfection of different siRNAs also found not cytotoxic to monocytes (90.5% live cells). No statistical significance was observed by One-way ANOVA analysis (P>0.05).

Cytotoxicity from the siRNA delivery was monitored by comparing viable cell numbers in cultures that were transfected with siRNA to that of non-transfected control. In this study, cytotoxicity was determined by measuring LDH released from cells with compromised membrane. Transfection experiment was optimized by treating the monocytes with increasing concentration of siRNA and transfection reagent. Results showed no evidence of toxicity regardless of the concentration of siRNA used and a mild toxicity with the increase of transfection reagent (data not shown). Furthermore, no cytotoxicity effect was observed at 72 h post-transfection of combined siRNAs in monocytes (>90% viable cells) (One-way ANOVA with Dunnett's post-test, P>0.05). This observation was further validated by counting the number of viable trypan blue stained cells under the microscope as shown in Figure 2.

### Quantification of infected cells and intracellular viral RNA load

**Figure 3:**
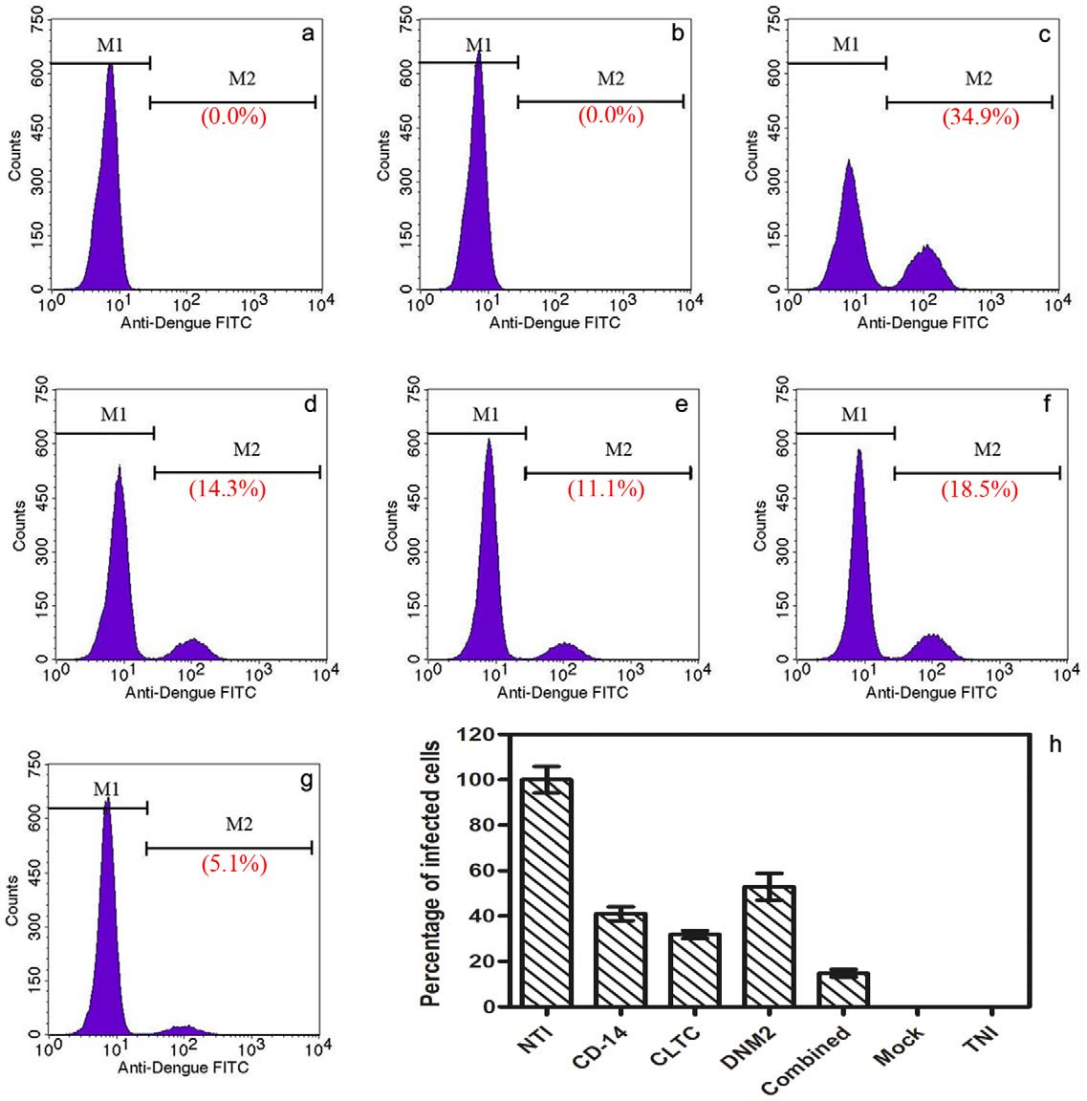
Quantification of infected cells by flow cytometry. At 72 h post-transfection, monocytes were infected by DENV-2 at MOI of 2. Marked reduction in percentage of infected cells was observed. This figure shows the percentage of DENV infected cells at different conditions. (a) Transfected non-infected monocytes (0.0%) represents the negative control. (b) Transfected mock-infected monocytes as a staining control (0.0%). (c) Non-transfected infected monocytes (34.9%) as a positive control. (d) CD-14 associated molecule silenced infected monocytes (14.3%). (e) CLTC silenced infected monocytes (11.1%). (f) DNM2 silenced infected monocytes (18.5%). (g) CD-14 associated molecule, CLTC, and DNM2 combined silenced infected monocytes (5.1%). (h) Summarized the results of the flow cytometry experiments. Data is expressed as a percentage of infected cells compared with non-transfected infected monocytes (NTI) which was defined as 100%. The percentages of the infected cells are 41.0%, 36.2%, 52.9% and 14.8% for CD-14 associated molecule, CLTC, DNM2, and combined silenced monocytes, respectively (One-way ANOVA with Dunnett's post-test, P<0.0001, Error bars are ± SD).

**Figure 4:**
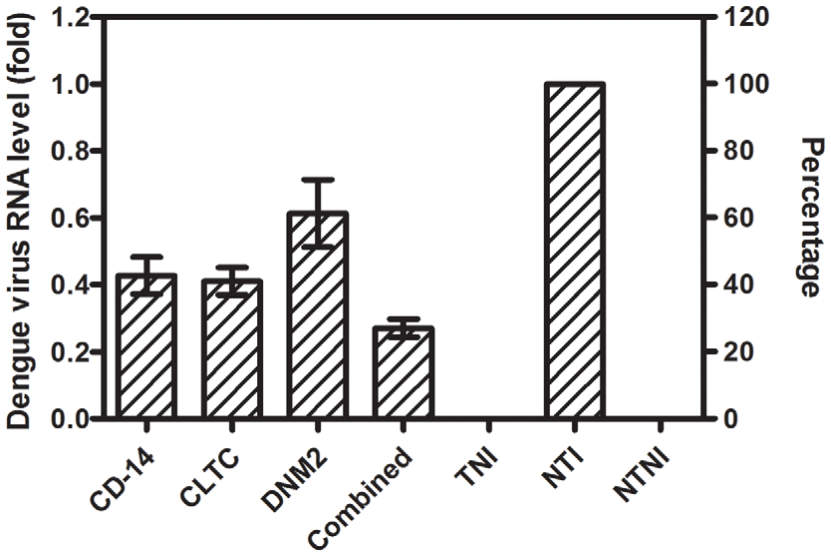
Intracellular dengue virus RNA load. Viral RNA levels were quantified by RT-QPCR and normalized to reference gene (RPL22). Data is expressed as relative fold expression to non-transfected infected monocytes control, which defined as 1.0 fold. Results show (0.57 fold±0.10), (0.59 fold±0.07), (0.39 fold±0.18), and (0.73 fold±0.05) reduction in viral RNA load in CD-14 associated molecule, CLTC, DNM2, and combined silenced monocytes, respectively (One-way ANOVA with Dunnett's post-test, P<0.0001, Error bars are ± SD). (TNI, Transfected Non-Infected; NTI, Non-Transfected Infected; NTNI, Non-Transfected Non-Infected).

Silenced and non-silenced monocytes were independently infected with DENV-2 at a MOI of 2 in order to determine whether silencing CD-14 associated molecule and/or clathrin endocytosis pathway could inhibit DENV entry into monocytes and, therefore, reduce its multiplication. We observed a marked reduction in the percentage of infected cells, CD-14 associated molecule, CLTC, and DNM2 silenced monocytes, by using flow cytometry. The percentage of infected cells was reduced from 34.9%±3.5 in non-transfected monocytes to 14.3%±0.4 (59.0%), 12.7%±0.3 (63.8%), and 18.5%±3.6 (47.1%) in CD-14 associated molecule, CLTC, and DNM2 silenced monocytes, respectively. Interestingly, combined silencing (CD-14 associated molecule, CLTC, and DNM2) of monocytes showed a higher inhibitory effect on DENV entry and replication (5.1%±0.6) which represented 85.2% reduction in the percentage of infected cells compared with non-silenced monocytes as shown in Figure 3 (One-way ANOVA with Dunnett's post-test, P<0.0001). This observation was further confirmed by RT-qPCR analysis. The viral RNA load in silenced monocytes was compared to the non-silenced monocytes and was normalized to the reference gene (RPL22). Data is expressed as relative fold expression to non-silenced monocytes, which was defined as 1.0 fold (viral RNA copy number is 3.35×10^3^/µl). Results showed a significant reduction of DENV RNA level in silenced monocytes: 0.57 fold±0.10, 0.59 fold±0.07, 0.39 fold±0.18, and 0.73 fold±0.05 in CD-14 associated molecule, CLTC, DNM2, and combined silenced monocytes, respectively as shown in Figure 4 (One-way ANOVA with Dunnett's post-test, P<0.0001).

### Quantification of culture supernatant viral RNA load

**Figure 5:**
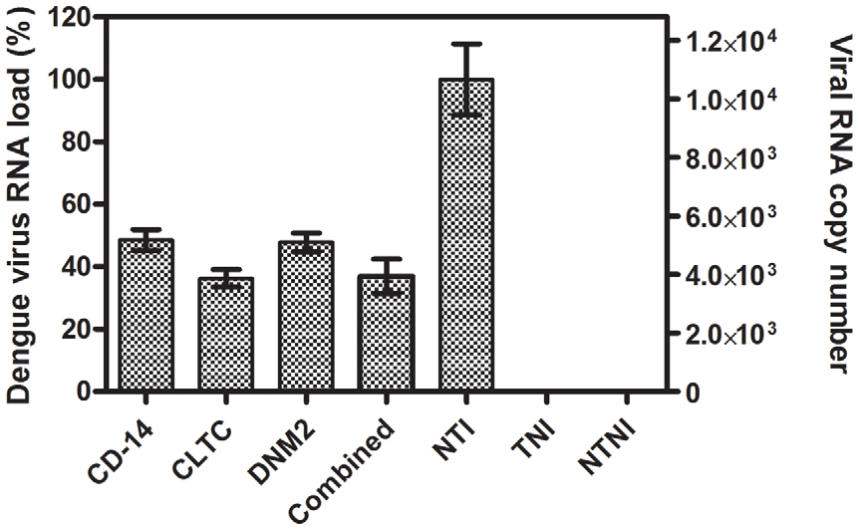
Extracellular dengue virus RNA load. DENV RNA in the culture supernatant of transfected and non-transfected monocytes was quantified by RT-qPCR. Result shows marked reduction in viral RNA (51.4%, 63.7%, 52.2%, and 63.0%) for CD-14 associated molecule, CLTC, DNM2, and combined silenced monocytes, respectively, when compared with non-transfected monocytes, which defined as 100% (viral RNA copy number is 1.06×10^4^/µl). This result is statistically significant (One-way ANOVA with Dunnett's post-test, P<0.0001, Error bars are ± SD). (TNI, Transfected Non-Infected; NTI, Non-Transfected Infected; NTNI, Non-Transfected Non-Infected).

DENV excreted from the silenced monocytes into the culture supernatant was titrated by RT-qPCR and compared to non-silenced monocytes. Results showed a similar observation when compared to the intracellular quantification results. Figure 5 described the DENV RNA load of the culture supernatant of silenced monocytes compared with non-silenced monocytes, which is defined as 100% (viral RNA copy number is 1.06×10^4^/µl). The reduction in viral RNA was 51.4%±3.4, 63.7%±2.9, 52.2%±3.0, and 63.0%±5.5 for CD-14 associated molecule, CLTC, DNM2, and combined silenced monocytes, respectively. This result is statistically significant (One-way ANOVA with Dunnett's post-test, P<0.0001).

## Discussion

We set off this study to determine the effect of RNAi in silencing the monocytes attachment receptor, and clathrin-mediated endocytosis to limit dengue infection. It was achieved through monocytes isolation from peripheral blood and infection with DENV-2 post siRNA transfection *in vitro* assay. Based on previous observations, one of the main target cell type for *in vivo* DENV infection is cells of the monocyte lineage [42]. It is responsible for the dissemination of viruses and the production of more viruses than other cells after its transmission via the mosquito vector [8], [9], [43], [44]. Macrophages are usually one of the first cell types to pick up inoculated virus at the site of entry but, the stage of which their differentiation is most susceptible to the virus entry is yet to be deduced. A number of viruses can replicate in monocytes and macrophages, and certain viruses replicate almost exclusively in monocyte lineage. Virological studies have recognized that both susceptibility to infection and viral gene expression increases during monocytes maturation to macrophages and is closely associated with persistence of latency of many viruses, such as cytomegalovirus [45], [46]. This shows that monocytes play an important role during dengue infection, progression and severity of the disease.

One of the biggest challenges of RNAi-based therapy is the efficient delivery of siRNAs to the target cells. Effective delivery of synthetic siRNAs requires elements that improve the pharmacological properties of the siRNA, and optimize all stages of the delivery process once the initial injection through the sequence-specific mRNA degradation in the cytoplasm of the target cells. Successful applications of RNAi in functional genomics, proteomics, and gene therapy depend on the efficiency of delivery of siRNA into mammalian cells [47]. Delivery approaches using cationic carriers, such as lipids, is the popular and practical method for siRNA delivery *in vitro*. However, for application of RNAi as a dengue therapeutic, an effective and cell-specific delivery system, *in vivo*, is required. Recently, several studies have described a success in siRNAs delivery, *in vivo*, by coupling to antibodies or peptides that recognize cell surface receptors [48]. This can provide a further supporting and future potential for the practical utility of this approach. Successful applications of RNAi in functional genomics, proteomics, and gene therapy depend on the efficiency of delivery of siRNA into mammalian cells [47]. The aim of any therapeutic is to maximize the ratio of desired effects to undesired effects. In some cases, such as chemotherapy, interferon treatment and highly active antiretroviral treatment, the ratio is not ideal and a significant degree of toxicity is associated with treatment. Although RNAi has the capacity to provide better gene targeting specificity, the exposure of cells to any exogenous molecule (siRNA or transfecting reagent) has the potential to disrupt normal cellular functions and needs to be carefully controlled. In this study, we optimized the transfection experiment that produced a maximum silencing effect at low-cell toxicity as revealed by measurement of LDH released from cells with compromised membrane and trypan blue dye in cell viability assay. We also found that a low-siRNA concentration is optimal and can achieve an efficient gene silencing. The approach of gene silencing is a widely accepted technique and, recently, RNAi technique had shown a potential to achieve the gene therapy goal [49]. There are several advantages of RNAi therapeutics, which include specific design targeted therapeutics for almost any gene regardless of the function of the gene product. RNAi mediated inhibition is more potent than that achieved with other antisense oligonucleotides and the versatility and ease with which RNAi mediated inhibition can induce multiple sequences within an individual gene, or a group of genes, can be targeted simultaneously and more readily providing the benefit of combination therapy [31]. Our results are in agreement with this point. We screened the effectiveness for three different designed siRNA pools targeting CD-14 associated molecule, CLTC and DNM2. Each pool achieved its own silencing level and consistent efficient knockdown of these genes in monocytes was achieved by co-transfection of the three pools simultaneously. The desire to pool three siRNAs was raised primarily from the finding that the silencing efficiency of a single siRNAs lesser than combined pool of triple siRNAs. This pools have shown a greater potency in the reduction of the target gene expression and elimination off-targets effect.

Focusing on the RNAi based therapeutic efficacy, one of the potential weaknesses, is the problem of resistance and RNAi escape mutations. This problem often results from using RNAi as an antiviral agent, as it has been demonstrated that both RNA and DNA viruses can quickly produce RNAi escape mutations [31]. This problem will probably necessitate the use of RNAi in combination therapy approaches, including multiple RNAi target sequences and/or other synergistic antiviral drugs. Another possible alternative way to overcome viral resistance is to provide a new therapeutic strategy by targeting the host factors known to be involved in viral infection, such as entry receptor or binding molecules on the susceptible cells. Thus, it is likely to suppress viral infection by making the cellular receptors for viruses on human cells less accessible. Dengue virus-host cell interaction is initiated with the virus binding on attachment receptors on the cell surface, followed by induction of signals that activate the endocytic internalization of the virus particle. This is required for successful entry into the cell and initiation of infection. This study was designed to target the susceptible receptor (CD-14 associated molecule), that assumed is responsible for attachment of DENV to monocytes [8], [16], [22] and clathrin-mediated endocytosis that known as the main pathway of viruses' internalization [22]. Recent studies showed that design and synthesis of agents that prevent DENV binding and entry to the cellular receptor sites could prove to be novel antiviral agents of preventing the disease. RNAi pathway shows a role in modulating DENV replication in different cells. It was observed that down regulation of Hsp60 in human monocytic myeloma cell line (U937) resulted in the decrease of percentage of infected cells [50]. Silencing the cellular genes involved in processes of endocytosis, cytoskeletal dynamics and endosome trafficking in Huh7 cells showed a potential inhibition of DENV [51], although these host targets weren't mentioned in the whole genome RNAi screens that have been published recently for West Nile and DENV [52]. Moreover, targeting the highly conserved sequence in the DENV envelope gene by siRNA effectively suppressed DENV replication in dendritic cells, macrophages [48] and C6/36 cells [53]. Significant inhibition of dengue infection is achieved by silencing the CD-14 associated molecule (up to 59%) and the clathrin-mediated endocytosis (up to 63.8%). Furthermore, more potent inhibition is reached (up to 85.2%) by combined silencing (CD-14 associated molecule, and clathrin-mediated endocytosis) simultaneously. This reduction in viral yield was not due to cell death as could be identified by cytotoxicity and viability tests (>90% live cells) in both transfected and non-transfected monocytes. Failure to achieve complete viral entry inhibition may be attributed to different reasons such as the use of alternative secondary receptors (such as Fc receptors and Hsp) or endocytosis pathways (such as phagocytosis and caveolae-mediated endocytosis) to establish the infection or incomplete inhibition by the RNAi machinery. For RNAi based therapeutics designing, it is necessary to consider that this technology knockdown gene expression, but in general does not eliminate it. Therefore, for some conditions, incomplete down regulation of a pathogenic gene seems to be adequate to produce clinically appropriate improvement [31]. The incidence of fatal DHF cases has been increased sharply especially in Asia over the last two decades, making it a leading cause of morbidity by inducing coagulopathy and vasculopathy [54]. One of the principal virological characteristics of severe DHF patients is the elevated viral load even during the defervescence stage [55]. This study suggests that, targeting the entry of DENV using RNAi is an attractive strategy for therapeutic intervention since it has a limit cell toxicity and attack the virus at the first step of the replication cycle. Moreover, another beneficial effect that could be expected is the avoidance of damages that could occur to the cells later during viral replication. Inevitably, reducing the entry of DENV into monocytes could be helpful in reducing the severity and incidence of the DHF by reducing the viremia during the infection course. Furthermore, this study confirms the previous findings of the role of CD-14 associated molecules and clathrin mediated endocytosis in DENV entry in the host cells and establishing the infection.

### Conclusions

We successfully inhibit the DENV entry and subsequent virus multiplication in monocytes by silencing the surface receptor and clathrin mediated endocytosis using RNA interference. This tool might serve as a novel promising therapeutic agent for the attenuation of dengue infection, which could subsequently leads to the reduction of disease transmission as well as progression to severe form of the disease DHF.

### List of accession numbers

NM_000591, NM_004859, NM_001005360, NM_001101, NM_000983, NM_001030001, NC001477.
